# Aerobic efficiency is associated with the improvement in maximal power output during acute hyperoxia

**DOI:** 10.14814/phy2.13119

**Published:** 2017-01-20

**Authors:** Tom A. Manselin, Olof Södergård, Filip J. Larsen, Peter Lindholm

**Affiliations:** ^1^ Swedish School of Sport and Health SciencesÅstrand Laboratory of Work PhysiologyStockholmSweden; ^2^ Department of Physiology and PharmacologyKarolinska Institute SwedenStockholmSweden

**Keywords:** Efficiency, exercise, hyperoxia, metabolism

## Abstract

This study investigated the relationship between aerobic efficiency during cycling exercise and the increase in physical performance with acute hyperoxic exposure (FiO2 ~31%) (HOX) and also tested the hypothesis that fat oxidation could be increased by acute hyperoxia. Fourteen males and four females were recruited for two sessions, where they exercised for 2 × 10 min at 100 W to determine efficiency. HOX and normoxia (NOX) were administered randomly on both occasions to account for differences in nitrogen exchange. Thereafter, a progressive ramp test was performed to determine *V*O_2max_ and maximal power output (*W*_max_). After 30 min rest, workload was set to 80% of maximal power output (*W*_max_) for a time to exhaustion test (TTE). At 100W gross efficiency was reduced from 19.4% during NOX to 18.9% during HOX (*P *≤* *0.0001). HOX increased fat oxidation at 100 W by 52% from 3.41 kcal min^‐1^ to 5.17 kcal min^‐1^ (*P *≤* *0.0001) with a corresponding reduction in carbohydrate oxidation. *W*_max_ increased by 2.4% from 388.8 (±82.1) during NOX to 397.8 (±83.5) during HOX (*P *≤* *0.0001). SaO_2_ was higher in HOX both at the end of the maximal exercise test and TTE. Subjects with a high level of efficiency in NOX had a larger improvement in Wmax with HOX, in agreement with the hypothesis that an optimum level of efficiency exists that maximizes power production. No association between mitochondrial excess capacity and endurance performance was found; increases in oxygen supply seemed to increase maximal aerobic power production and maintain/increase endurance capacity at the same relative workload.

## Introduction

For a number of decades it has been known that exercise performance may be improved by the breathing of hyperoxic gases, the rationale behind this intervention being that more oxygen is delivered to the working tissues, both by an increase in the amount of dissolved oxygen in the blood and also by an increase in the saturation of hemoglobin. These effects are made evident by the abolished exercise‐induced arterial hypoxemia (EIAH) experienced in some individuals and the increased maximal oxygen consumption (*V*O_2max_) shown during severe exercise (Powers et al. 1989) Since EIAH is more pronounced in well‐trained individuals than those who are more sedentary, it is reasonable to assume that the former group experiences a larger benefit from hyperoxic breathing. However, this hypothesis is not proven and needs to be investigated in well‐controlled studies.

Early reports indicated that maximal oxygen consumption (*V*O_2max_) increases by 5–18% (for review see (Astorino and Robergs 2003)), while exercise performance increases markedly less (around 4–8%) after hyperoxic breathing. A methodological obstacle to accurate determination of aerobic efficiency and *V*O_2max_ during hyperoxic exposure is that oxygen uptake is difficult to measure, due to non‐negligible nitrogen exchange across the body. This problem is especially marked when breathing gases with >50% O_2_ content. Despite these complications, the reported dissociation between increases in *V*O_2max_ and increases in performance could indicate that aerobic efficiency is hampered by hyperoxic exposure, a finding that is supported by some (Welch and Pedersen 1981; Prieur et al. 2002) but not all studies (Byrnes et al. 1984).

Aerobic efficiency, also referred to as gross efficiency, is defined as the work output to metabolic input ratio, and can be measured using indirect calorimetry and a cycle ergometer. In traditional exercise physiology, it has been shown that among subjects with similar *V*O_2max_, those with higher more type I muscle fibers and higher aerobic efficiency have a higher level of physical performance (Horowitz et al.^1994^). From a naive perspective it might seem logical that higher efficiency is always desirable because that would increase the work output for a given metabolic input. However, from a thermodynamic view, increases in efficiency comes with the trade‐off of lower reaction rates and 100% efficient conditions, and no heat loss are forbidden according to the second law of thermodynamics. Therefore, for every external condition there must be a level of efficiency where the combination of efficiency and rate of the reaction gives a maximum power output (Aledo and del Valle 2004). This has been termed the optimal efficiency that maximizes power production (OEMP). At the OEMP, both increases and decreases in efficiency must lead to lower power production.

Changes in efficiency could therefore be either positive or negative, depending on the subjects’ initial level of efficiency. If efficiency decreases due to acute oxygen exposure, this could counteract or enhance the beneficial effects of the increased oxygen supply on exercise performance. Likewise, in pathological conditions with reduced oxygen availability, reductions in efficiency can be found (Richardson et al. 2004) and should be regarded as positive adaptations to allow a higher power production.

The increased *V*O_2_ at a given absolute workload during hyperoxic exposure could be explained by a substrate switch from carbohydrate toward fat. It is well known that fat oxidation is hampered in hypoxia (Roberts et al. 1996); Lundby and Van Hall 2002); therefore, it is conceivable that hyperoxia could increase fat oxidation. This notion is supported by studies that show a decreased glycogenolysis and lower lactate production (Stellingwerff et al. 2005, 2006) indicating greater fat oxidation while breathing 60% oxygen during exercise. A greater reliance on fat as a substrate could increase endurance performance and spare glycogen stores. However, the contribution of altered substrate utilization to the increased exercise performance has not been assessed previously, neither has fat oxidation been quantified in absolute terms during submaximal exercise while breathing hyperoxic gas.

In order for any increases in oxygen supply to have a beneficial effect, the mitochondrial capacity must be in excess of the capacity of the heart to deliver oxygen to the tissue. The functional relevance of having a mitochondrial excess capacity is debated, but a widely accepted hypothesis is that it is important for endurance performance and fat oxidation (Gollnick and Saltin 1982). This is supported by the fact that well‐trained subjects have a larger excess capacity than untrained subjects (Gifford et al. 2016). However, the hypothesis has proven difficult to test mechanistically. If exercise is performed at the same relative workload in normoxia and acute hyperoxia, the excess mitochondrial capacity becomes less in hyperoxia, due to the increased oxygen supply rate. In this study, we wanted to test this theory by investigating endurance performance (time to exhaustion) at the same relative workload in normoxia and hyperoxia.

The intent of this study was to recruit subjects with a large range of aerobic capacity and use a novel, single‐blinded, double cross‐over design to account for differences in nitrogen exchange with hyperoxic breathing. The primary aim was to investigate how changes in aerobic efficiency contributed to changes in performance under HOX. Further objectives were to test if fat oxidation was increased by acute administration of HOX and if time to exhaustion at the same relative but higher absolute work rate was changed by HOX.

## Methods

### Subjects

Eighteen healthy subjects, 14 males (age 31.6 (±6.1) years; height 183.5 (±6.2) cm; weight 82.7 (±10.6 kg), BMI 24.5 (±2.7) kg/m^2^) and four females (27.5 (±5.4) years; 163.8 (±6.1) cm; 60.2 (±6.8) kg, BMI 22.4 (±1.3) kg/m^2^), volunteered for the study. The subjects classified themselves in terms of fitness, ranging from moderately active up to elite. The study was performed in conformity with the Declaration of Helsinki and approved by the local Ethics Committee. Written, informed consent was obtained from each subject prior to inclusion in the study. Initially, 20 subjects were recruited, but two were excluded at an early stage: one subject was excluded from data analysis due to inability to complete the 100 W exercise at a steady state, while the other only performed one test session due to an upper respiratory tract infection.

### Experimental protocol (submax, ramp, and TTE)

This study was designed as a randomized, cross‐over, single‐blind experimental trial. Subjects visited the laboratory on two occasions, with a minimum of 5 and a maximum of 14 days apart. Body weight and height were measured before the tests. Subjects were randomized to start the tests breathing either a hyperoxic gas (31% O_2_) (HOX) or a normoxic gas (NOX), with air as the placebo. To control for non‐negligible nitrogen exchange (i.e., the release of tissue and blood nitrogen when breathing HOX), subjects performed the submaximal exercise both with NOX and HOX on both visits, once with NOX immediately followed by HOX, and on the other with HOX first and then NOX. If any release of N2 did occur, this would be evident as a difference in *V*O_2_, due to the order of the administration of the gases. However, we did not observe any order effect between the different conditions (*V*O_2_ was elevated with 72 ± 3 mL with HOX first and 78 ± 5 mL with NOX first, *P *=* *0.63). Therefore, results are presented as the mean of the two visits.

### Submaximal test

To allow more time for equilibration of gases in the tissue, subjects breathed gas from Douglas bags filled either with NOX or HOX gas. After 5 min of equilibration, the subjects exercised for 2 × 10 min, at a workload of 100 W with a cadence of 50 rpm. This first submaximal test was randomized, so that half of subjects started the first 10 min with NOX and half with HOX. Expired gas was collected for analyses in Douglas Bags for 2 × 90 sec after 5 min on each condition (HOX/NOX), and capillary blood samples for blood lactate measurements were taken.

### Maximal exercise test

After a short rest period, a progressive ramp test was performed. The starting load was adjusted after subjective evaluation of the fitness of the subject (many were athletes with known data). Load increased stepwise by 25 W after each full minute for males and 20 W per min for females. On the days where the subjects breathed NOX during the max exercise, test *V*O_2_ was calculated by collecting expired gas with a Jaeger Oxycon Pro (CareFusion, GmbH, Hoechberg, Germany) in mixing chamber mode. During HOX breathing, oxygen was added to the inspired room air via a facemask adjusted to supply O_2_boluses from an Oxelerate system (Oxelerate, Version 1.1 with firmware Version 1.24, Oxelerate AB, Drottningholm, Sweden). Expired gas was not collected during HOX breathing due to technical difficulties in collecting gas from that particular design of face mask.

### Time to exhaustion test

Finally, after 30 min of rest, the workload was set to 80% of the maximal power output (*W*_max_) achieved in the preceding maximal test and the subjects made a “time to exhaustion test” while breathing the same gas as during the progressive ramp. The calculation of *W*_max_ was as follows:

$$W_{\max}=W_{f}+\text{[(t/60)}\times 25)]$$

*W*_f_ is the value of the last completed workload (W), t is the time of the last uncompleted workload (s), 60 is the duration of each completed workload (s), and 25 is the power output difference between each workload (Padilla et al. 1999).

The subjects were fully informed as to the experimental design, but were blinded to the order of the Douglas bag supplied gas, which was randomly assigned and equally distributed among them. The use of the Oxelerator in the progressive ramp test could not be blinded and so some subjects were aware that they were breathing hyperoxic gas. However, they were always blinded to their performance and the duration of the ramp, as well as their performance in the time to exhaustion test.

### Materials and equipment

All tests and training interventions were performed at the Laboratory of Applied Sports Science (LTIV) of the Swedish School of Sport and Health Sciences (GIH) in a climate‐controlled room. The temperature and humidity were set to 20°C and 50%, respectively, throughout the test.

The tests were conducted on a SRM Ergometer (Stationary cycle ergometer *SRM, GmbH, Jülich, Germany*). In order to record the power output data, the ergometer was connected to a computer (PC) via an USB‐cable. The data from the cycle ergometer were logged every second and included power output (W), cadence (rpm), and heart rate (bpm).

To administer HOX or NOX to the subjects during the steady‐state test, Douglas bags were prefilled with either the hyperoxic gas mixture or air from 10 L gas tanks. Oxygen concentration in the tanks was analyzed and ranged from 30.07% to 32.9%.

The oxygen for the ramp and the time to exhaustion tests was produced with a slightly modified oxygen concentrator (NewLife Intensity, Airsep Corp, Buffalo) and intermittently supplied through an Oxelerator. A difference in pressure allowed the Oxelerator to detect when the subjects started their inspiration, and then added oxygen at a flow of 18 L per minute and a valve timing of 0.5 sec during the inspiratory phase. Since the quantity of oxygen added during the inspiration cycle was constant, the FIO_2_ varied with tidal volume. Thus, a higher tidal volume inferred a lower FIO_2_. The flow data were recorded on a PC with Oxelerate software (Version 2.5, Oxelerate AB, Tumba, Sverige), which was also used to adjust the settings on the Oxelerator. During HOX, the inspiratory oxygen concentration level (FiO_2_) was 26–30% when mixed during inspiration with room air.

Gas analysis for calculations of *V*O_2_ etc. during the steady‐state test were made with the Douglas bag method (Douglas 1911) using a CO_2_ Analyzer, Gold Edition, Model 17515 (Vacumed, Ventura), and an O_2_ Analyzer Gold Edition, Model 17518 (Vacumed).

During the ramp test with air, the exhaust gas was analyzed with a Jaeger Oxycon Pro. Measurements included oxygen uptake (*V*O_2_), carbon dioxide production (*V*CO_2_), respiratory exchange ratio (RER), ventilation (VE), breathing frequency (BF), expired oxygen fraction (FEO_2_), and expired carbon dioxide fraction (FECO_2_). Heart rate and SpO_2_ were measured continuously with a Radical‐7 Signal Extraction Pulse CO‐Oximeter (Masimo Corporation, Irvine) attached to the left earlobe. Data were recorded through a MP150 Data Acquisition System (BIOPAC Systems, Inc, Goleta) using AcqKnowledge 4.1.1 software (BIOPAC Systems). Heart rate was also monitored by a Polar^™^ H2 heart rate monitor (Polar Electro Oy, Kempele, Finland).

For all lactate and glucose measurements, a Biosen C‐Line Clinic (*EKF‐diagnostics, GmbH, Barleben, Germany*) was used to analyze the samples. A blood capillary micro‐sample (20 *μ*L) was collected from the fingertip and then put in prefilled reaction cups containing a hemolyzing solution of 1 mL (*EKF safe‐lock*). Samples were obtained before the start of the tests (resting lactate), during the last minute at each sub maximal level (both in normoxia and hyperoxia), as well as on 1 min and 3 min after cessation of the maximal work and the TTE tests, giving a total of seven samples.

### Statistical analysis

Results are expressed as the mean ± SEM. Student's *t*‐test was used for statistical analysis (software: Graphpad Prism 5.0) between NOX and HOX. Pearson r test was used for correlative findings. *P *<* *0.05 was considered as significant. D'Agostino‐Pearson omnibus normality test was used to test for normal distribution of the data.

## Results

### Aerobic efficiency and substrate oxidation

**Figure 1:**
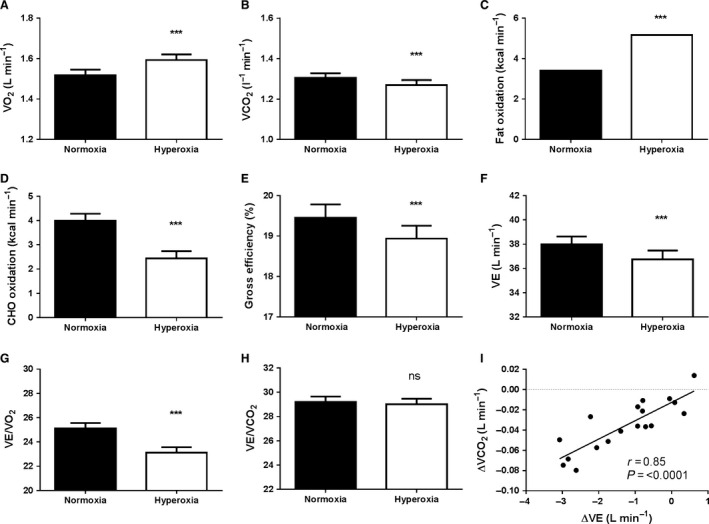
Hyperoxia increases fat oxidation while decreasing efficiency during submaximal exercise. Subjects cycled at 100 watts and a cadence of 50 rpm while respiratory parameters were measured by the Douglas bag technique. To control for time‐dependent drift of oxygen consumption and non‐negligible nitrogen exchange, the values presented are averages of two independent tests; one where normoxia were followed by hyperoxia and a second test where hyperoxia were followed by normoxia. (A) is the pulmonary oxygen uptake, (B) is the volume of exhaled CO_2_, (C) is the absolute quantification of fat oxidation, (D) is the absolute quantification of carbohydrate oxidation, and (E) is gross efficiency calculated as work output/metabolic energy expenditure. (F) is pulmonary ventilation (L min^−1^), (G) VE/VO_2_ is liter pulmonary ventilation per liter oxygen uptake, (H) VE/*V*CO_2_ is liter pulmonary ventilation per liter exhaled carbon dioxide, (I) is the correlation between the change in pulmonary ventilation and change in volume of exhaled carbon dioxide. ***P <* *0.001

At the 100 W submaximal workload, *V*O_2_ was higher in HOX (1.593 L min^−1^) compared to NOX (1.518 L min^−1^) (*P *≤* *0.0001), see Figure 1A. *V*CO_2_ was reduced in HOX (1.269 L min^−1^) compared to NOX (1.304 L min^−1^) (*P *≤* *0.0001), see Figure 1B.

After calculating the thermic equivalent from different ratios of substrate oxidation according to Mansell and Macdonald (1990), fat oxidation was increased by 52% from (3.41 kcal min^−1^) in NOX to (5.17 kcal min^−1^) in HOX (*P *≤* *0.0001). Carbohydrate oxidation was reduced from (4.00 kcal min^−1^) *V*in NOX to (2.44 kcal min^−1^) in HOX (*P *≤* *0.0001), respectively, see Figure 1C+D.

Fat oxidation requires more oxygen per unit energy than carbohydrate oxidation. The fact that we found a markedly higher fat oxidation after HOX could explain why we also found a higher *V*O_2_ after HOX. When calculating gross efficiency (work output/energy input) this was taken to account. Nevertheless, gross efficiency was reduced from 19.4% during NOX to 18.9% during HOX, (*P *≤* *0.0001), see Figure 1E.

Despite the lower gross efficiency, pulmonary ventilation (VE) was reduced from 38.0+/−0.6 in NOX to 36.7+/−0.7 L min^−1^ in HOX, see Figure 1F. VE/*V*O_2_ was therefore also reduced while VE/*V*CO_2_ was unchanged, see Figure 1G–H. Interestingly, we found a very close relationship between the change in *V*CO_2_ and VE (*R* = 0.85, *P *≤* *0.0001, Fig. 1I) confirming that the ventilatory drive mainly is controlled by the CO_2_ levels. The reduced *V*CO_2_ can directly be related to the increased fat oxidation which yields less metabolic CO_2_ than carbohydrate oxidation and thereby attenuating the ventilatory drive (Lindholm et al. 2007).

### Maximal exercise test

Subjects *V*O_2max_ during the ramp test during NOX ranged from 2.83 to 6.69 L min^−1^ with a mean (SD) of 4.33(±1.0) L min^−1^. Corresponding data related to weight were 43.8–71.3 with a mean (SD) of 55.8 (9.0) mL kg^−1^ min^−1^. Thus, subjects with a wide range of physical fitness were included in the group.

**Figure 2:**
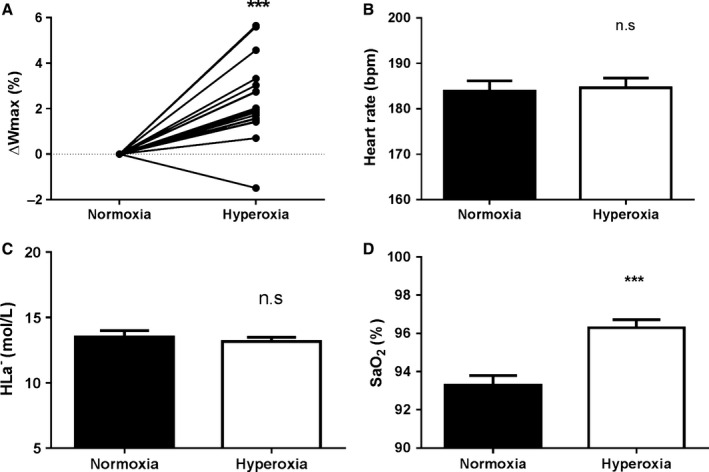
Hyperoxia increases maximal attainable power and arterial oxygen saturation. (A) denotes the change in maximal attainable power during incremental exercise to exhaustion in hyperoxia compared to normoxia, (B) is the maximal heart rate at exhaustion, (C) is maximal capillary lactate concentrations 3 min after completion of the incremental test, and (D) is the arterial oxygen saturation measured in an earlobe during the last minute of the incremental test. ***P <* *0.001

Maximal power production increased by 2.4% during the progressive ramp test from 388.8 (±82.1) W during NOX to 397.8 (±83.5) W during HOX, *n* = 18, (*P *≤* *0.0001), Figure 2A.

There was no significant difference in maximal heart rate (NOX 184 ± 10 and HOX 185 ± 9 bpm, Figure 2B). Lactate values at 3 min post exercise were 13.5 (±2.15) in NOX and 13.16 (1.43) in HOX, *n* = 18, NS, Figure 2C. SaO_2_ was elevated at the end of the maximal exercise test from NOX 93.3 (±2.1) versus HOX with 96.3% (±1.7), *n* = 18, (*P *≤* *0.0001), Figure 2D.

### Time to exhaustion

**Figure 3:**
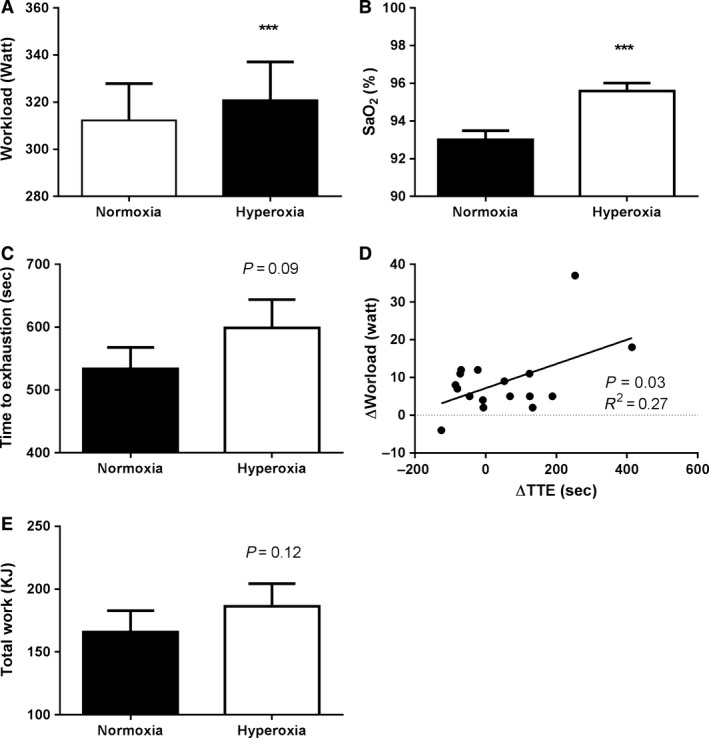
Performance at the same relative workload is unchanged in hyperoxia despite working at a higher absolute workload. Subjects were asked to cycle until exhaustion at 80% of the *W*_max_ in both normoxia and hyperoxia. Despite cycling at a higher absolute workload in hyperoxia than normoxia (A), with arterial oxygen saturation that was higher in hyperoxia (B), time to exhaustion was unchanged between conditions (C). A closer analysis revealed a significant relationship between the increase in workload and the increase in time to exhaustion (D). (E) A trend toward more work performed was seen in HOX. ***P <* *0.001

While administration of HOX acutely increased maximal power production, HOX is very unlikely to increase mitochondrial capacity, this postulate that the subjects utilized a higher fraction of their mitochondrial capacity in HOX. To test how this affected endurance exercise capacity, we measured time to exhaustion (TTE), where subjects exercised at 80% of peak power achieved at the preceding maximal test both in NOX and HOX. The average workload was 311.5(±68.1) W with NOX and 320.3 (±71.0) W with HOX, *P *=* *0.001, Figure 3A. SaO_2_ was higher during the endurance test in HOX at 95.7 (1.5)% versus NOX 93.1(2.0), *n* = 16, (*P *=* *0.0002), Figure 3B. Maximal Lactate (La) did not differ between conditions at 1 nor 3 min post TTE. The highest values were measured after 1 min with 12.98 (1.85) in HOX and 13.32 (2.53) in NOX, *n* = 16, NS, data not shown.

**Figure 4:**
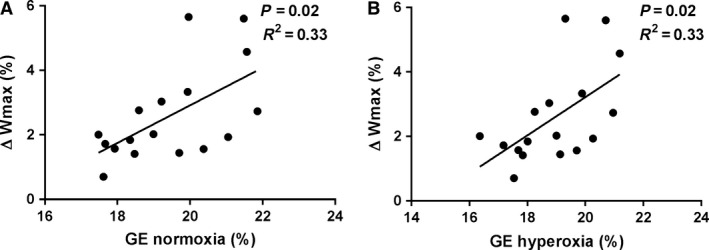
Baseline gross efficiency predicts the improvement in maximal power production during incremental exercise. Correlation between gross efficiency in (A) normoxia, (B) hyperoxia, and the increase in maximal power production. One subject out of 18 had a lower maximal power production in hyperoxia and was excluded from the analysis.

Surprisingly, despite the higher workload and therefore higher fractional utilization of the mitochondrial capacity with HOX, the TTE was 521.1(±137.6) s with NOX and 570.9(±152.5) s with HOX, but the differences were not significantly different (*P *=* *0.09, Fig. 3C). Upon subanalysis, we correlated individual changes in workload during the endurance test with changes in TTE, and a significant positive association between the change in TTE and change in workload was found (*r* = 0.52, *P *=* *0.03, Fig. 3d). When total work was calculated, a similar pattern as for TTE was seen with a trend toward more work performed in HOX (*P *=* *0.12, see Fig. 3E). Together, these findings suggest that the improved performance by HOX primarily is mediated by increase in maximal aerobic power production and that the effect of a higher oxygen supply overrides any potentially negative peripheral effects from forcing the muscles to work at a higher absolute work rate.

### Baseline aerobic efficiency predicts the improvement in maximal power production during hyperoxia

To gain a deeper understanding behind the mechanism that improves performance during HOX, we ran correlation analyses between improvements in *W*max and *V*O_2max_, SaO_2_, and gross efficiency. Unexpectedly, we found that neither baseline *V*O_2max_ (*r* = 0.13, NS) nor changes in SaO_2_ (*r* = 0.04, NS) correlated with improvements in *W*max (data not shown). However, gross efficiency both in NOX and HOX did correlate with improvements in Wmax (*r* = 0.44 and 0.45, respectively, both *P *=* *0.06). When the only subject that showed an impaired performance in HOX was excluded from the analysis, this relationship was substantially increased (*r* = 0.59, *P *=* *0.01 and *r* = 0.57, *P *=* *0.02), respectively (Fig. 4).

## Discussion

It has been known for many decades that breathing of hyperoxic gases might improve performance. However, identifying individuals who respond well to hyperoxic interventions have proven difficult. We here show that subjects with high aerobic efficiency at baseline tend to improve their maximum power output more than subjects with lower efficiency.

Furthermore, we demonstrate that fat oxidation is markedly increased and carbohydrate oxidation decreased with HOX. We have not been able to find any previous studies in the literature that have quantified fat oxidation in absolute terms during hyperoxia; these findings support the hypothesis that fat oxidation is highly sensitive to changes in oxygen availability and could explain the glycogen‐sparing effect previously found with hyperoxic exercise (Stellingwerff et al. 2005). The increased reliance of fat as a substrate could theoretically enhance endurance performance; however, the endurance test in this study was too brief (~9 min) to gain any real advantage from increased fat oxidation and sparing of glycogen reserves. To detect any positive effect of an increased ability to oxidize fat, the endurance test has to last long enough, probably more than 90 min, to allow for a significant energy contribution from lipid sources.

The switch toward a greater oxidation of fat occurs quickly, and is detectable after only a few minutes of breathing HOX and is reversed as quickly when NOX is administered again. The mechanistic basis behind the switch in substrate utilization is unknown but physiological responses that occur within such a short time frame disallow major protein modifications or mitochondrial alterations to occur. The effect is more likely mediated by acute oxygen sensing mechanisms or by changes in metabolite accumulation in the same fashion as fat oxidation is hampered at high exercise intensities (Harris et al. 1987). A critical factor in fat oxidation is the availability of free carnitine (van Loon et al. 2001). Carnitine associates with long‐chain fatty acids and is mandatory for their transport into the mitochondria. Indeed, as exercise intensity increases, and fat oxidation decreases, the muscle content of free carnitine decreases and the content of acetylcarnitine increases (Harris et al. 1987; Sahlin 1990).

A speculative, but not unlikely, explanation is that it is the lower efficiency per se that allows the higher rate of fat oxidation in this study. The lower efficiency mandates a higher flux through the TCA cycle, and thus a high rate of incorporation of acetyl groups with CoA. In this manner, the TCA cycle “steals” acetyl groups and uses it for oxidative phosphorylation that otherwise would react with carnitine to form acetylcarnitine, making the carnitine group unavailable for fatty acid transport. It has been proposed that the transport of fatty acids into mitochondria potentially can limit fat oxidation (Sahlin 2009); this allows even a small decrease in efficiency such as that found in this study (‐2.5%) to liberate enough carnitine to allow the marked 54% increase in fat oxidation that we found at submaximal work.

The decrease in gross efficiency during HOX is interesting and resembles our previous findings when exposing subjects acutely to a hypoxic gas (16% O_2_), where we also found acute decreases in gross efficiency (Schiffer et al. 2014). In the same article, we found that mitochondrial efficiency, is directly dependent on oxygen availability such that the mitochondrial P/O ratio was impaired at elevated oxygen levels. Our results are in agreement with earlier reports in animal tissues showing higher mitochondrial efficiency in hypoxia (Gnaiger et al. 2000). Further evidence of oxygen availability as a modulator of efficiency is the recent discovery of two isoforms of cytochrome c oxidase subunit IV in human skeletal muscle (Schiffer et al. 2016). It was shown that subjects with a higher proportion of the COX IV‐2 isoform had lower basal metabolic rate and cells overexpressing the COX IV‐2 isoform had higher mitochondrial efficiency. It has previously been suggested that the COX IV‐2 isoform is induced by hypoxia while COX IV‐1 is prevalent at higher oxygen tensions (Fukuda et al. 2007).

Interestingly, in this study, we found that the initial level of efficiency correlated with the increase in Wmax, such that subjects with high initial efficiency were the ones who showed the most robust increase in Wmax after HOX. This finding fits well with the theory that an optimal level of efficiency exists, which allows for maximal power production (Odum and Pinkerton 1955; Schiffer et al. 2014). It is likely, therefore, that some subjects had a level of efficiency that was slightly higher than the level that maximizes power production, and thus showed a larger increase in Wmax as efficiency was lowered by HOX.

Since mitochondrial capacity is unlikely to change with hyperoxic breathing, mitochondrial excess capacity was decreased by acutely increasing SaO_2,_ and thus oxygen supply, via administration of an hyperoxic gas. The subjects worked at the same relative work rate (80% of *W*_max_) but at a higher absolute work rate in HOX (320.3 ± 71.0 W) in comparison to NOX (311.5 ± 68.1 W). Although the excess mitochondrial capacity was decreased by breathing the HOX, there was a trend for TTE to increase during HOX, but this was not statistically significant. The positive association between increases in workload and increases in TTE suggest that individuals who responded well to the intervention and could increase their *W*_max_, also increased their TTE despite having to work at a higher absolute workload. These results indicate that the increases in oxygen supply is the primary factor that dictates increases in Wmax and that the extra muscular stress from increasing the absolute workload from 311 to 320 W is negligible if the relative workload is kept constant. The question remains as to what the functional role of having such a large mitochondrial overcapacity is? A possible explanation could be that an excess capacity provides a buffer against oxidative insults, as it has been shown recently that short‐term high intensity training can inhibit mitochondrial respiration through oxidative inactivation of aconitase (Larsen et al. 2016).

An alternative explanation is that the mitochondrial capacity is indeed increased by the hyperoxic exposure. Generally, mitochondrial respiration is assumed to be independent of oxygen availability under physiological conditions. However, we have recently measured mitochondrial oxygen affinity (p50_mito_) in the human skeletal muscle, and found it to be around 0.04 KPa, that is, high enough to have a small, but significant regulatory role under physiological conditions (Larsen et al. 2011). Indeed, a previous study found that cellular PO_2_ during maximal exercise increased from ~0.4 when breathing 21% oxygen to ~0.55 KPa with 100% oxygen (Richardson et al. 1999). The relationship between the cellular oxygen pressure (PO_2_), P50_mito_, and the relative activation of mitochondrial Vmax (J'max) is described by the equation:

$$j^{\prime }O_{2}=\frac{pO_{2}}{\left(pO_{2}+p50\text{mito}\right)}$$

At a cellular PO_2_ of 0.4 KPa and a mitochondrial p50mito of 0.04 KPa, mitochondria respire at 0.4/(0.4 + 0.04) = 90.9% of their *V*_max_ due to oxygen conformance, that is, is inhibited by 10% due to the nonsaturating oxygen levels. If the PO_2_ is increased to 0.55 KPa, oxygen conformance is diminished and mitochondria can then respire at 0.5/(0.5 + 0.04) = 92.6% of *V*_max_. This 1.7% increase in oxygen uptake could make a significant contribution to the performance outcome in this study. Interestingly, we have found that aerobic efficiency correlates positively with p50_mito,_ such that subjects with high aerobic efficiency have a high p50_mito_. Thus, subjects with high aerobic efficiency and a high p50_mito_ will experience a more severe oxygen conformance at any PO_2_, and will at least benefit mathematically more from an increased PO_2_ with hyperoxic breathing.

For the athletic community, our results can identify individuals who will experience a larger performance gain with hyperoxic training that theoretically could alter training adaptations (Perry et al. 2007). Based on our observations, this identification can be done by simply measuring gross efficiency in normoxia; subjects with higher efficiency will experience larger acute performance increases.

Furthermore, the increase in fat oxidation is rather dramatic; a 52% increase at 100 watts might have big impact on both weight loss and could potentially increase the effectiveness of exercise interventions in clinical populations including obesity, diabetes, and metabolic syndrome.

In conclusion, we have shown that breathing a moderately (~30% O_2_) hyperoxic gas has substantial effects on substrate oxidation, maximal aerobic power, and aerobic efficiency. It was shown that subjects with a high efficiency seem to respond better to HOX than subjects with lower efficiency.

## Conflict of Interest

Author P. Lindholm hold 25% of the patent rights for the oxelerator apparatus.
